# CD154 and IL-2 Signaling of CD4^+^ T Cells Play a Critical Role in Multiple Phases of CD8^+^ CTL Responses Following Adenovirus Vaccination

**DOI:** 10.1371/journal.pone.0047004

**Published:** 2012-10-05

**Authors:** Channakeshava Sokke Umeshappa, Roopa Hebbandi Nanjundappa, Yufeng Xie, Andrew Freywald, Yulin Deng, Hong Ma, Jim Xiang

**Affiliations:** 1 Cancer Research Unit, Saskatchewan Cancer Agency, University of Saskatchewan, Saskatoon, Saskatchewan, Canada; 2 Departments of Oncology and Pathology, University of Saskatchewan, Saskatoon, Saskatchewan, Canada; 3 Beijing Institute of Technology, Beijing, China; University of Colorado Denver, United States of America

## Abstract

Adenoviral (AdV) vectors represent most commonly utilized viral vaccines in clinical studies. While the role of CD8^+^ cytotoxic T lymphocyte (CTL) responses in mediating AdV-induced protection is well understood, the involvement of CD4^+^ T cell-provided signals in the development of functional CD8^+^ CTL responses remain unclear. To explore CD4^+^ T helper signals required for AdVova-stimulated CTL responses, we established an adoptive transfer system by transferring CD4^+^ T cells derived from various knock out and transgenic mice into wild-type and/or CD4-deficient animals, followed by immunizing with recombinant ovalbumin (OVA)-expressing AdVova vector. Without CD4^+^ T help, both primary and memory CTL responses were greatly reduced in this model, and were associated with increased PD-1 expression. The provision of OVA-specific CD4^+^ T help in CD4^+^ T cell-deficient mice restored AdVova-induced primary CTL responses, and supported survival and recall responses of AdVova-stimulated memory CTLs. These effects were specifically mediated by CD4^+^ T cell-produced IL-2 and CD154 signals. Adoptive transfer of “helped” or “unhelped” effector and memory CTLs into naïve CD4^+^ T cell-deficient or -sufficient mice also revealed an additional role for polyclonal CD4^+^ T cell environment in the survival of AdVova-stimulated CTLs, partially explaining the extension of CTL contraction phase. Finally, during recall responses, CD4^+^ T cell environment, particularly involving memory CD4^+^ T cells, greatly enhanced expansion of memory CTLs. Collectively, our data strongly suggest a critical role for CD4^+^ T help in multiple phases of AdV-stimulated CTL responses, and could partially explain certain failures in AdV-based immunization trials targeting malignant tumors and chronic diseases that are often associated with compromised CD4^+^ T cell population and function.

## Introduction

CD8^+^ T cells play a defensive role against viral infections and malignancies. Following recognition of a specific antigen (Ag), naïve CD8^+^ T cells undergo 3 distinct phases [1]: (i) a proliferation (or primary) phase in which naïve CD8^+^ T cells undergo autonomous clonal expansion and develop into functional effector cytotoxic T lymphocytes (CTLs) [2], [3]; (ii) an effector phase in which effector CTLs clear the invaded pathogen and about 90–95% of effector pool undergo activation-induced cell death through apoptosis, allowing ∼5–10% of the initial population to develop into memory CTLs; and (iii) a maintenance (or memory) phase in which memory CTLs survive for a prolonged duration. Upon subsequent Ag encounter, memory CTLs respond swiftly by rapid proliferation and heightened effector functions.

It is becoming increasingly clear that requirements for CD4^+^ T cell help at different phases of CTL responses can vary, depending on a specific type of infection or immunization involved [4], [5]. Primary CTL responses to infectious agents, such as *Listeria montocytogenes* (Lm), influenza and Lymphocytic choriomeningitis virus (LCMV), occur independent of CD4^+^ T-helper signals [6]–[8]. In contrast, primary CTL responses induced in noninfectious conditions by minor Ags, and cell-associated and protein-triggered immunizations [9], [10], and also CTL responses in infectious diseases, such as Herpes simplex (HSV), Viral encephalitis and Vaccinia virus [4], [11]–[13], heavily depend on CD4^+^ T cell signals. Requirement for cognate CD4^+^ T cell signals during priming in functional memory CTL development has been frequently suggested [7], [8], [10], [11], [14]. It has been shown that signaling induced by CD4^+^ T cell-expressed CD154 is needed for the generation of memory CTLs in the course of the Lm, LCMV and influenza infections [15]–[18]. In relation to AdV-induced immunity, Yang et al initially observed the importance of CD4^+^ T cells for primary CTL responses to AdV immunization [19], [20]. Subsequently, others also showed the importance of CD4^+^ T cells for AdV-specific primary CTL expansion [21]–[23]. However, the role of CD4^+^ T cells in priming that modulate secondary CTL responses is still controversial. Yang *et al* [21], [24] reported that CTLs generated in CD4^+^ T cell-deficient environment were less functional, while yet retaining their proliferating ability during recall responses. In contrast, Holst *et al* [22] showed the generation of dysfunctional memory CTLs that neither provided the protection against a lethal virus challenge nor retained the ability to proliferate during recall responses in the absence of CD4^+^ T cells. In addition, Mu et al [23] reported that the respiratory mucosal route of AdV immunization precluded CD4^+^ T helper dependence for effective CTL responses. The molecular mechanisms involved in CD4^+^ T-helper effects during the primary responses that control different stages of CTL engagement are still unknown. Furthermore, the exact contributions of CD4^+^ T cells during maintenance and recall phases to CTL survival and expansion are yet to be determined. In this study, we addressed these issues by systematically investigating the requirement for CD4^+^ T cells in multiple phases of ovalbumin (OVA)-specific CD8^+^ CTL responses induced by vaccination with recombinant OVA-expressing adenovirus (AdVova).

## Results

### AdVova Stimulates Persistent CD4^+^ and CD8^+^ T Cell Responses

**Figure 1:**
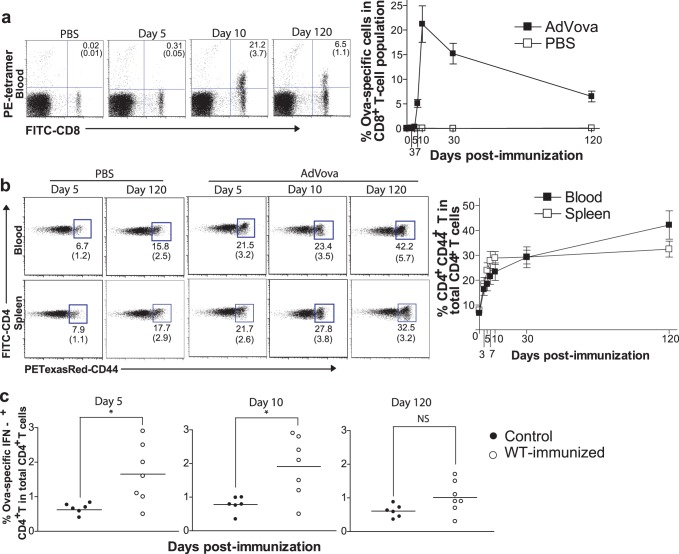
AdVova stimulates persistent CD4^+^ and CD8^+^ T cell responses. Following immunization, the AdVova-specific CTLs (**a**) and CD44^+^CD4^+^ T cells (**b**) in the peripheral blood and/or spleens were analyzed by flow cytometry at the indicated time points after tetramer staining, and CD44 and CD4 double marker staining, respectively. The values in the figure (left panel) or line diagram (right panel) are presented as mean%±SD of OVA-specific CD8^+^ CTLs in total CD8^+^ T cell population (**a**) or of CD44^+^ CD4^+^ T cells in total CD4^+^ T cell population (**b**), and are cumulative of three independent studies with three to five mice per group. (**c**) Following immunization, the spleen samples were analyzed for OVA-specific CD4^+^ T cells by intracellular IFN-γ staining at the indicated intervals. The values (% frequencies) are cumulative of two independent experiments with three to four mice per group. *P<0.05, versus matching controls.

Initially, we assessed the kinetics of CD4^+^ and CD8^+^ T cell responses, at different time points following AdVova vaccination, by staining cells in mouse blood with FITC-CD4/nonspecific PE-CD44 or FITC-CD8/OVA-specific PE-tetramer, respectively, followed by flow cytometric analysis. We observed a peak of OVA-specific CD8^+^ T cell responses on day 10 (21.2±3.7% of total CD8^+^ CTLs) and a long-term maintenance of high levels of OVA-specific CTLs (as much as 6.5±1.1% of total CD8^+^ CTLs) even 4 months after AdVova immunization (Fig. 1a), indicating that, in line with earlier reports [21], [25], CD8^+^ CTLs persist for a prolonged period of time. In addition, our experiments also showed that AdVova-stimulated CD4^+^ T cells persist in both peripheral blood and spleen. In contrast to CTL population that showed greater than 60% contraction 4 months later, AdVova-stimulated poly-specific CD4^+^ T cells gradually increased their levels with time, representing 21.5±3.2% and 23.4±3.5% of total blood CD4^+^ T cells as early as on 5 and 10 days post-immunization, respectively, and 42.2±5.7% in 4 months (Fig. 1b). Their higher levels were also maintained up to 4 months in the spleen. This increase was mainly due to AdVova vaccination rather than to an old age, as we observed only modest increase in levels of CD4^+^CD44^+^ T cells (15.8±2.5% of total blood and 17.7±2.9% of total spleen CD4^+^ T cells) in control PBS-injected mice sacrificed 4 months after the treatment. In addition, we also demonstrated that the frequency of OVA-specific, IFN-γ-secreting CD4^+^ T cells is significantly higher in the immunized mice than in control animals on 5^th^ and 10^th^ day after immunization (P<0.05) (Fig. 1c). However, frequency increase was statistically insignificant in immunized mice sacrificed on the day 120 following the immunization. This diminished transgene OVA-specific CD4^+^ T cell response could be due to the predominance of other AdV vector-specific CD4^+^ T cell responses as recently described in the human model [26]. Nevertheless, taken together, these results suggest that AdVova immunization also triggers prolonged transgene-specific, and likely, AdV vector-specific CD4^+^ T cell responses.

**Table 1:** Molecular mechanisms of CD4^+^ T-helper signals required for functional AdV-specific memory CTL responses^a^.

AdV_OVA_ immunization	% Tumor-bearing mice	Tumor metastasis grading
PBS	8/8 (100)	++++++
WT	0/12 (0)	–
MHCII^−/−^	12/12 (100)	+++++
MHCII^−/−^+polyCD4^+^ T	10/10 (100)	+
MHCII^−/−^+polyCD4(IL-2^−/−^)	10/10 (100)	+++++
MHCII^−/−^+polyCD4(CD154^−/−^)	10/10 (100)	++++
MHCII^−/−^+polyCD4(CD80^−/−^)	10/10 (100)	++
MHCII^−/−^+OTIICD4^+^ T	6/10 (60)	−/+
MHCII^−/−^+OTIICD4(CD154^−/−^)	10/10 (100)	+++
MHCII^−/−^+OTIICD4(CD80^−/−^)	10/10 (100)	+

a One day prior to immunization, MHCII^−/−^ mice were adoptively transferred with CD11c^+^ DCs (∼0.5–1.0×10^6^/mouse) and naïve polyclonal (∼15–20×10^6^/mouse) or OTII CD4^+^ T cells (∼1.5×10^6^/mouse) with or without designated gene deficiency, as indicated. 120 days later, all the immunized mice were challenged with BL6-10ova tumor cells. Twenty-four days after the challenge, lung tumor colonies were counted and graded. The data are cumulative of two independent experiments, each comprising five to six mice per group.

### CD4^+^ T Cells Influence the Kinetics of AdVova-specific CTL Responses

**Figure 2:**
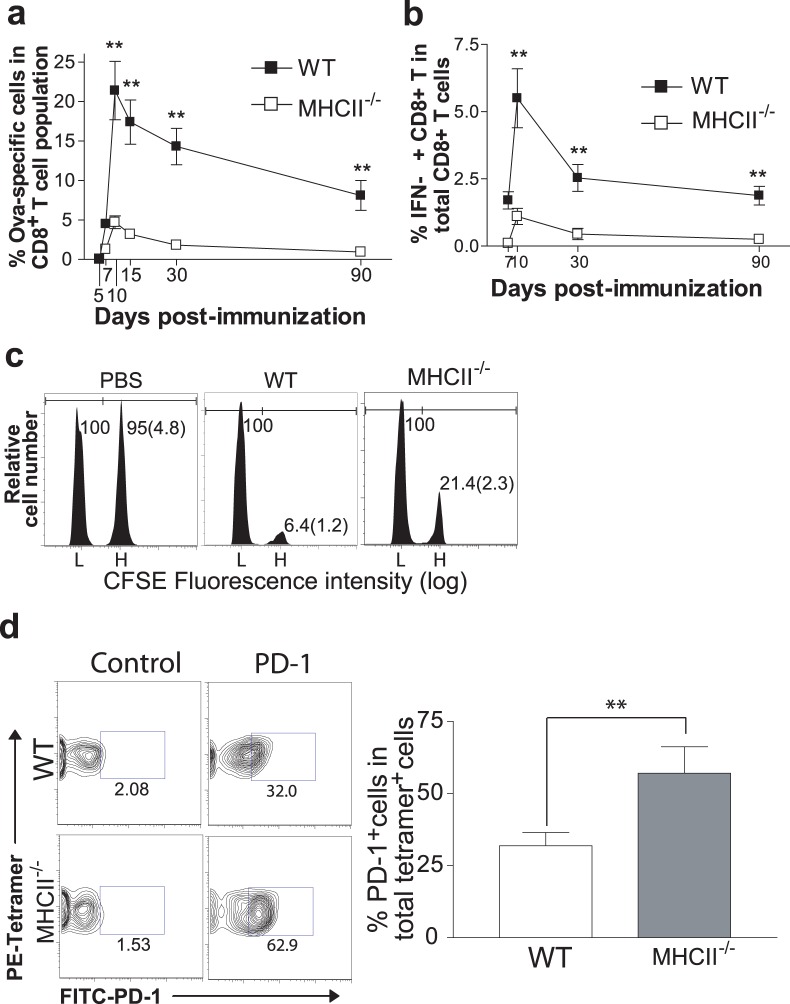
CD4^+^ T cells impact the kinetics of AdV transgene-specific CTL populations. Following immunization, AdVova-specific CTLs were analyzed in the peripheral blood at different time points by tetramer (**a**) and intracellular IFN-γ (**b**) stainings. The values are presented as mean%±SD of OVA-specific tetramer^+^ CTLs (**a**) or IFN-γ^+^ CTLs (**b**) in total CD8^+^ T cell population and are representative of two to three independent experiments with three to four mice per group. **P<0.01, versus MHCII^−/−^ mice. (**c**) Ten days following immunization, the proportions of CFSE^high^-OVAI-pulsed target cells lysed by effector CTLs were determined in the spleens by *in vivo* cytotoxicity assay. The values represent mean %±SD of targets remaining in spleens relative to controls and are representative of two independent experiments with three to four mice per group. (**d**) On day 75, following immunization, OVA-specific memory CTLs were characterized in spleen for PD-1 expression by flow cytometry. A representative figure from immunized groups along with matching isotype control is shown on the left. The values in the bar diagram represent the mean %±SD of PD-1^+^ tetramer^+^ CTLs in total tetramer^+^ CTL population and are representative of two independent experiments with 3 to 4 mice per group. **P<0.01, versus WT mice.

To assess the influence of CD4^+^ T cells in AdVova-stimulated CTL responses, we immunized major histocompatibility complex class II deficient (MHCII^−/−^) mice lacking CD4^+^ T cells with AdVova and analyzed OVA-specific CD8^+^ CTL responses at different time points by flow cytometry. In agreement with previous observations [21], we found 4 to 6 fold decrease in the primary CTL proliferation in MHCII^−/−^ compared to WT mice (Fig. 2a). Furthermore, 90 days after immunization, WT mice had ten-fold higher percentages of CTLs compared to MHCII^−/−^ mice. Although primary CTLs in MHCII^−/−^ mice (unhelped CTLs) expanded less efficiently, they still appeared to be functional, and retained IFN-γ-secreting ability (Fig. 2b) and cytotoxic functions (Fig. 2c). Considering relatively prolonged persistence of AdVova in the body, it is possible that CD4^+^ T cells could influence the exhaustion of AdVova-specific CTLs. To assess this possibility, we monitored the expression of PD-1, a well-established exhaustion marker, on helped and unhelped CTLs [27]. Indeed, MHCII^−/−^ mice showed considerably higher percentages of PD-1-expressing memory CTLs in spleens, compared to WT mice on day 75 post-immunization (Fig. 2d). Although significantly less overall PD-1-expression (by approximately 3 fold) could be observed during the effector phase, once again, we detected nearly 3-fold reduction in the PD-1 expression in CTLs from WT versus MHCII^−/−^ mice (data not shown). This indicates that potential differences in viral persistency in WT compared to MHCII^−/−^ mice are unlikely to influence CTL exhaustion. Overall, our data strongly suggest that CD4^+^ T cells enhance AdV-stimulated CTL survival and rescue them from exhaustion by inhibiting PD-1 expression.

### CD4^+^ T Cell-derived CD154 and IL-2 Signaling in AdVova-specific Primary and Memory CTL Responses

**Figure 3:**
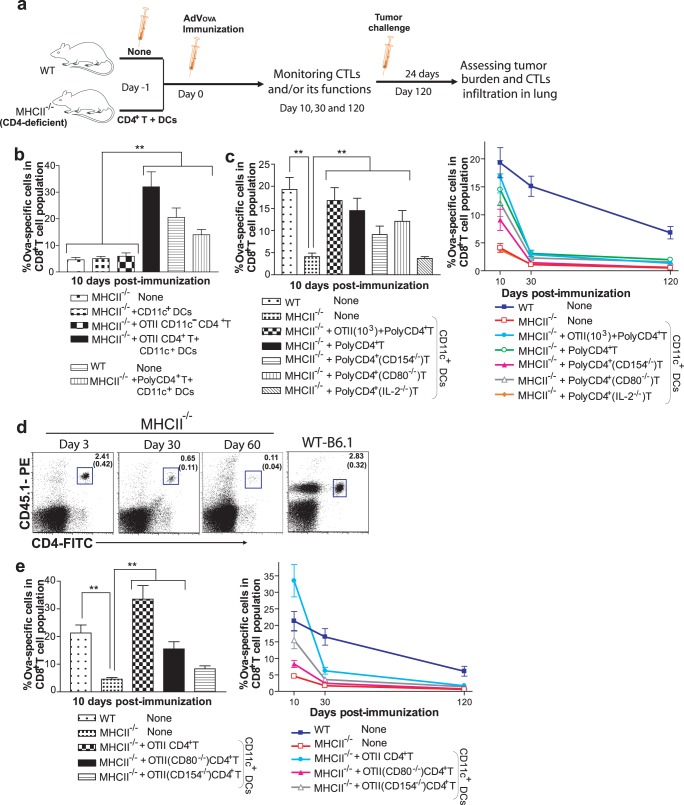
The molecular mechanisms of CD4^+^ T cell help in CTL primary responses and survival. (**a**) A schematic protocol. MHCII^−/−^ or WT mice were adoptively transferred with monoclonal (∼1.5×10^6^/mouse) or polyclonal CD4^+^ T cells (∼15–20×10^6^/mouse) with or without designated gene deficiency and supplied with spleen-derived CD11c^+^ DCs (∼0.5–1.0×10^6^/mouse). One day later, both MHCII^−/−^ and WT mice were i.v. immunized with AdVova followed by assessment of CTL proliferation in the peripheral blood at the indicated intervals. After 120 days (memory stage) following the immunization, all the groups were challenged with BL6-10_OVA_ tumor cells and assessed for tumor protection. (**b**) Optimizing CD4^+^ T cells dose required for CTL expansion in MHCII^−/−^ mice. MHCII^−/−^ mice were transferred with monoclonal (OTII) and/or polyclonal CD4^+^ T cells and CD11c^+^ DCs, as shown in the figure. One day later, all the groups were immunized and assessed for CTL proliferation by tetramer assay. The values represent mean %±SD of OVA-specific tetramer^+^ CTLs in total CD8^+^ T cell population and are representative of two independent experiments with three to four mice per group. **P<0.01, versus MHCII^−/−^ mice with no adoptive CD4^+^ T cell transfer. (**c**) Molecular mechanisms of CD4^+^ T cell help. MHCII^−/−^ mice were transferred with polyclonal CD4^+^ T cells with or without designated gene deficiency and CD11c^+^ DCs as indicated. One day later, all the groups were immunized and subsequently assessed for CTL proliferation by tetramer assay. (**d**) Poor survival of adoptively transferred naïve CD4^+^ T cells in MHCII^−/−^ mice. Naïve CD4^+^ T cells (∼15×10^6^/mouse) on CD45.1^+^ background were transferred to congenic MHCII^−/−^ mice (CD45.2^+^) and analyzed 3, 30 and 60 days later by flow cytometry. One representative figure of a group is shown in the dot plot. The values in dot plots represent mean±(SD)% of two independent experiments, each comprising two to three mice per group. (**e**) Molecular mechanisms of CD4^+^ T cell help. MHCII^−/−^ mice were transferred with monoclonal (OTII) CD4^+^ T cells with or without designated gene deficiency and CD11c^+^ DCs as indicated. One day later, all the groups were immunized and subsequently assessed for CTL proliferation by tetramer assay. In (**c**) and (**e**), the values represent mean %±SD of OVA-specific tetramer^+^ CTLs in total CD8^+^ T cell population on day 10 post-immunization (left panel) or at the indicated time points (right panel) and are representative of two independent experiments with five to six mice per group. **P<0.01, versus MHCII^−/−^ mice with no adoptive CD4^+^ T cell transfer.

To assess the molecular mechanisms of CD4^+^ T cell-provided help, we developed an immunization protocol with a combination of AdVova immunization and adoptive transfer of polyclonal or cognate TCR transgenic OTII CD4^+^ T cells (OTII T cells) in MHCII^−/−^ mice (Fig. 3a). Since AdVs persists for a relatively longer period, an environment, which completely lack CD4^+^ T cells for the prolonged duration of the study, would represent an ideal experimental model. This prompted us to choose MHCII^−/−^ mice for studying the role of CD4^+^ T cells in AdVova vaccination. Indeed, these animals have been extensively used to delineate the role of CD4^+^ T cells in AdV and other viral infections or tumor models [11], [28]–[30]. On the other hand, antibody-mediated depletion of CD4^+^ T cells is not only impracticable for the prolonged duration of the study, but also might confound interpretation of the results since it also depletes immunosuppressive Tregs. This is in contrast to the situation in MHCII^−/−^ mice, which are known to retain functional subsets of CD4^+^ Tregs [29], [31]. We first determined the optimal dose of purified monoclonal or polyclonal CD4^+^ T cells required for efficient CTL responses (data not shown). Supplying polyclonal CD4^+^ T (∼15–20×10^6^) or OTII T cells (∼1.5×10^6^) cells was found to be efficient in recovering primary CTL response in MHCII^−/−^ to the level observed in WT mice (Fig. 3b). Since Ag-presenting cells (APCs) in these animals lack the MHCII molecules and are unable to stimulate transferred CD4^+^ T cells, spleen-derived CD11c^+^ DCs (∼1×10^6^) were also required for the adoptive transfer to initiate AdVova-stimulated CTL responses. Indeed, the transfer of polyclonal or OTII T cells alone failed to enhance CTL responses, and the critical roles of CD11c^+^ DCs in AdV immunization have been well demonstrated previously [32], [33]. In the presence of polyclonal CD4^+^ T cell help, the primary CTL expansion was considerably increased in MHCII^−/−^ mice, most probably because of the existence of endogenous precursor CD4^+^ T cells. Strikingly, the primary CD8^+^ CTLs expanded even more efficiently in the presence of OTII CD4^+^ T cells in MHCII^−/−^ than in WT mice (1.5 fold difference), indicating that this immunization protocol can be used to assess the molecular mechanisms of CD4^+^ T cell help, when CD4^+^ T cells, derived from IL-2-, CD154- and CD80-deficient mice, are transferred.

To address the involvement of specific molecular factors, we reconstituted MHCII^−/−^ mice with polyclonal or monoclonal (OTII) CD4^+^ T cells with or without a deficiency in selected genes and with CD11c^+^ DCs one day prior to the immunization. Once again, the reconstitution of polyclonal CD4^+^ T cells in MHCII^−/−^ mice strongly supported primary CTL expansion, compared to WT levels, and the CTL response was further increased, when endogenous levels of cognate (OTII) CD4^+^ T cells were additionally provided (Fig. 3c). Strikingly, the primary expansion was severely impaired in the absence of IL-2 signaling by polyclonal CD4^+^ T cells, strongly resembling the situation in MHCII^−/−^ mice. In addition, the primary expansion was significantly reduced, when polyclonal CD4^+^ T cells lacking CD154 (*P*<0.01) were transferred, while the absence of CD80 did not produce any noticeable negative effect. During the memory phase, CTL survival significantly decreased in MHCII^−/−^ compared to WT mice (*P*<0.01) irrespective of CD4^+^ T cell transfer (Fig. 3c, right panel). This reduction is most probably due to the progressive loss of transferred CD4^+^ T cells in MHC-II-deficient environment [34]. We observed greater than 75% and 95% reduction of transferred naïve CD4^+^ T cells at days 30 and 60 of post-adoptive transfer in unimmunized MHCII^−/−^ mice (Fig. 3d). Nevertheless, MHCII^−/−^ mice reconstituted with polyclonal CD4^+^ with or without OTII T cells had considerably higher frequencies of CTLs, when compared to MHCII^−/−^ mice alone or to MHCII^−/−^ mice transferred with polyclonal CD4^+^ T cells lacking CD154 or IL-2 (*P*<0.01). Strikingly, the transfer of cognate CD4^+^ T cells (∼1.5×10^6^) supported the primary CTL expansion, surpassing the WT levels (Fig. 3e). In contrast, the absence of costimulatory molecules, particularly CD154, suppressed the ability of cognate CD4^+^ T cells to support primary CTL expansion, mimicking the response in untreated MHCII^−/−^ mice. Interestingly, polyclonal or OTII CD4^+^ T cells without CD154 signaling did support primary CTL expansion in MHCII^−/−^ mice to some extent when compared to the complete absence of CD4^+^ T cells, suggesting a possible role for other helper molecules presented by CD4^+^ T cells. As previously, CTLs survived poorly in MHCII^−/−^ mice during the memory phase, irrespective of OTII T cell transfer. Nevertheless, MHCII^−/−^ mice reconstituted with OTII T cells with or without CD80 had relatively higher frequencies of effector CTLs, when compared to MHCII^−/−^ alone (*P*<0.01) or to MHCII^−/−^ animals reconstituted with CD154-deficient polyclonal CD4^+^ T cells (*P*<0.05) (Fig. 3e, left panel).

To study the impact of CD4^+^ T cell-generated signals during the priming for functional memory CTL responses, the mice were challenged 120 days later with highly metastasizing, OVA-expressing mouse melanoma tumor cells. WT, but not MHCII^−/−^ animals were completely protected against tumors (Table 1). However, MHCII^−/−^ mice which received CD4^+^ T-helper-produced signals during priming got protected to various extents, depending on signaling content. Although MHCII^−/−^ mice with transferred polyclonal CD4^+^ T cells developed tumors, they had very low tumor burdens compared to mice receiving CD154-deficient or IL-2-deficient polyclonal CD4^+^ T cells. Interestingly, 40% of the challenged MHCII^−/−^ mice that were transferred with OTII T cells, showed antitumor protection. In contrast, mice transferred with CD154-deficient OTII T cells remained completely unprotected. Taken together, these data highlight the importance of cognate CD4^+^ T-helper signals, specifically CD154 and IL-2, in AdV immunization, in particular in the optimization of the primary expansion, and functional memory development.

### Naïve Polyclonal CD4^+^ T Cells Support Maintenance of AdVova Transgene-OVA-specific CTLs

**Figure 4:**
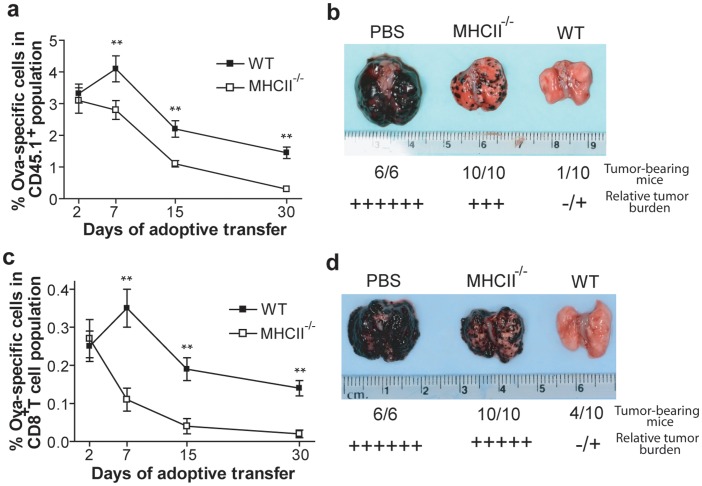
Polyclonal CD4^+^ T cells support maintenance of AdVova transgene product-specific CTLs. (**a**) Ten days following immunization, total CD8^+^ CTLs containing AdVova-specific effector CTLs were purified from B6.1 mice (CD45.1^+^ background) and adoptively transferred to naïve congenic WT and MHCII^−/−^ mice (CD45.2^+^ background; ∼15×10^6^/mouse). The OVA-specific tetramer^+^ CTLs were tracked after staining peripheral blood samples with tetramer reagent and congenic marker up to 30 days post-adoptive transfer. The values represent mean %±SD of OVA-specific tetramer^+^ CTLs in total CD45.1^+^ adoptively transferred T cell population at the indicated intervals (right panel) and are representative of two independent experiments with five to six mice per group. **P<0.01, versus MHCII^−/−^ mice. (**b**) Forty-five days after adoptive transfer, the above mice groups were challenged with BL6-10_OVA._ Twenty-four days after the challenge, both groups were assessed for tumor protection. Images represent distorted pathology of lungs, showing relative surface tumor burden. (**c**) Ninety days following the immunization, total CD8^+^ T cells containing AdVova-specific memory CTLs were purified from WT mice and adoptively transferred into naïve WT and MHCII^−/−^ mice (∼15×10^6^/mouse). The OVA-specific tetramer^+^ CTLs were tracked in peripheral blood samples by tetramer staining. The values represent mean %±SD of OVA-specific tetramer^+^ CTLs in total CD8^+^ T cell population at the indicated intervals (right panel) and are representative of two independent experiments with four to six mice per group. **P<0.01, versus MHCII^−/−^ mice. (**d**) Thirty days after the adoptive transfer, the above mice groups (**c**) were challenged with BL6-10_OVA_ and the relative surface tumor burden was assessed 24 days after the challenge as detailed above.

The observed faster decrease in the frequency of CTLs in MHCII^−/−^ compared to WT mice, even in the presence of reconstituted CD4^+^ T-helper population during priming (Fig. 3c and 3e), suggests a possible role for polyclonal CD4^+^ T cell environment in the maintenance of CTLs after priming. To examine this possibility, the effector CTLs from B6.1 mice (CD45.1^+^) were purified on day 10 following AdVova immunization and transferred into naïve congenic WT or MHCII^−/−^ mice (CD45.2^+^) and tracked by the tetramer and congenic marker staining. Consistent with our previous observations (Fig. 2a, 2b, 3c or 3e), the effector CTLs declined drastically starting from day 7 in MHCII^−/−^ mice, while a significantly slower decline was characteristic for WT mice (Fig. 4a). The possibility of CD4^+^ T cell stimulation in the WT recipient from the adoptive transferred cells was not ruled out in the current study. However, we believe there is a little possibility as most of the APCs expressing OVAI and OVAII peptides are likely removed during the purification of CD8^+^ T cells. Nevertheless, the relative contributions of cognate versus polyclonal CD4^+^ T cell help needs further investigation, as both types of help appear to be required for the survival of OVA-specific CTLs following the effector phase. In agreement, WT mice showed nearly complete protection against tumor challenge, while MHCII^−/−^ mice remained practically unprotected (Fig. 4b). Furthermore, memory CTLs purified after 90 days following immunization behaved similar to effector CTLs, showing reduced survival rate in MHCII^−/−^ mice (Fig. 4c). Again, WT animals received much better protection, when compared to MHCII^−/−^ mice (Fig. 4d). In summary, these observations suggest that polyclonal CD4^+^ T cell environment may be required for optimal maintenance of AdVova-specific CTLs.

### CD4^+^ T Cell Signals Provided during the Priming and Recall Phases are Required for Optimal Secondary Responses

**Figure 5:**
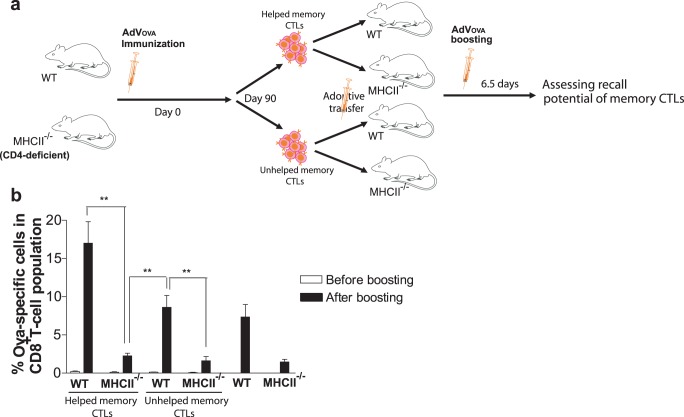
CD4^+^ T cell signals provided during priming and recall phase are required for optimal secondary responses. (**a**) A schematic protocol. After 90 days of immunization, total CD8^+^ T cells containing memory CTLs were purified from WT B6 mice with helped CTLs or MHCII^−/−^ mice with unhelped CTLs, adoptively transferred in equal numbers into the naïve secondary recipients, WT and MHCII^−/−^ mice (∼15×10^6^/mouse), and assessed for recall potential after boosting. (**b**) Three days after adoptive transfer of helped or unhelped memory CTLs into naïve WT and MHCII^−/−^ mice, all the mice groups were boosted with AdVova and monitored for the expansion of memory CTLs 6.5 days later. The values represent mean %±SD of OVA-specific tetramer^+^ CTLs in total CD8^+^ T cell population and are representative of two independent experiments with five to six mice per group. **P<0.01, versus MHCII^−/−^ mice with helped or unhelped memory CTLs.

Recent studies in multiple models have shown that CD4^+^ T cells are essential for optimal recall responses [1], [35]–[37]. The results of our experiments (Fig. 3c and 3e) also suggest the role for cognate CD4^+^ T cell signals, partly produced during priming, for functional memory responses, thus supporting previously published data [7], [8]. To further elucidate the relative importance of CD4^+^ T cells in primary and/or recall responses, memory CTLs were purified from splenocytes of WT B6 mice with helped CTLs or MHCII^−/−^ mice with unhelped CTLs after 90 days of AdVova immunization and adoptively transferred in equal quantities to the naïve secondary recipients, WT or MHCII^−/−^ mice (Fig. 5a). Following AdVova boosting, helped memory CTLs robustly expanded in WT when compared to their expansion in MHCII^−/−^ mice (Fig. 5b). Curiously, unhelped memory CTLs in WT showed increase in the expansion only slightly above the levels observed in WT without Tm transfer. Moreover, unhelped memory CTLs in MHCII^−/−^ mice failed to expand and showed levels similar to MHCII^−/−^ mice without Tm cell transfer. These results suggest possible synergistic role for CD4^+^ T cell help in both priming and recall responses.

**Figure 6:**
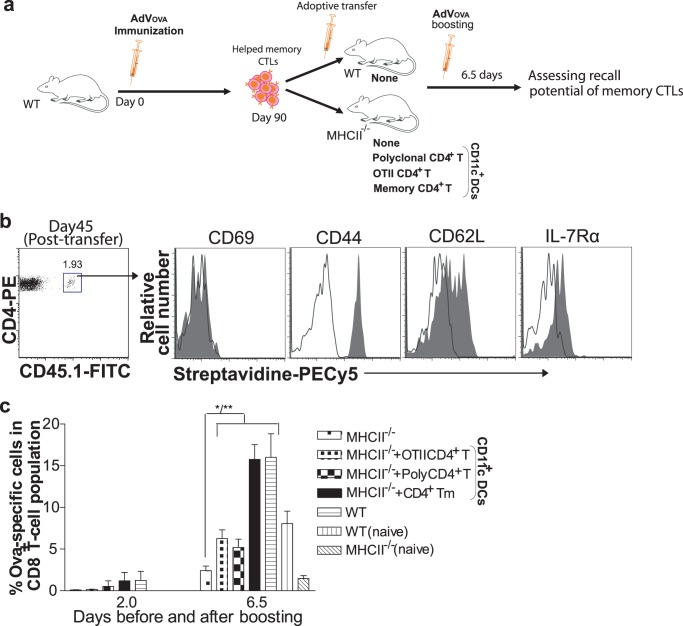
CD4^+^ T cell signals delivered during priming and recall phase are required for optimal secondary responses. (**a**) A schematic protocol. After 90 days following immunization, total CD8^+^ T cells containing memory CTLs were purified from WT (helped memory CTLs) mice and adoptively transferred equally to the naïve secondary recipients, WT and MHCII^−/−^ mice (∼15×10^6^/mouse). The MHCII^−/−^ mice were additionally reconstituted with different types of CD4^+^ T cells (∼15–20×10^6^/mouse) along with CD11c^+^ DCs (∼0.5–1.0×10^6^/mouse). Both groups were boosted with AdVova and assessed for memory CTLs expansion. (**b**) Naïve B6.1/OTII CD4^+^ T cells were co-cultured with irradiated BM DCova, as detailed in material and methods to generate Th1 cells. Th1 cells were then transferred to naïve congenic WT mice (∼10–15×10^6^/mouse). After 45 days, these cells were triple stained and phenotypically characterized for the expression of activation or memory markers (grey-shaded area) as shown in the figure. Irrelevant isotype-matched Abs were used as control (dotted thin lines). The value in the dot plot indicates % of OVA-specific CD4^+^ T memory cells remaining in total CD4^+^ T cell population. One representative of the two independent experiments is shown. (**c**) Naïve MHCII^−/−^ mice were adoptively transferred with helped memory CTLs with naïve OTII T cells (∼1.5×10^6^/mouse), polyclonal CD4^+^ T cells (∼15–20×10^6^/mouse) or polyclonal CD4^+^ T cells containing OVA-specific memory CD4^+^ T cells (∼15–20×10^6^/mouse) and CD11c^+^ DCs (∼0.5–1.0×10^6^/mouse) as shown in Fig. 6a. Three days later, all the groups were boosted with AdVova and the recall potential of the memory CTLs were assessed 6.5 days later. The values represent mean %±SD of OVA-specific tetramer^+^ CTLs in total CD8^+^ T cell population and are representative of two independent experiments with four to five mice per group. *P<0.05 or **P<0.01, versus MHCII^−/−^ mice with helped memory CTLs alone.

To further understand the nature of CD4^+^ T cell helper during recall responses, the adoptive transfer technology was employed again in MHCII^−/−^ mice (Fig. 6a). Here, MHCII^−/−^ mice, were transferred with CD11c^+^ DCs and OTII cells, polyclonal CD4^+^ T cells, or OVA-specific memory CD4^+^ T cells after being adoptively transferred with helped memory CTLs (Fig. 6b) as shown in Fig. 6a. As expected, helped CTLs in the presence of CD4^+^ T cells produced a modest increase in the expansion, but the response was considerably much higher in the presence of memory CD4^+^ T cells (Fig. 6c). On the other hand, MHCII^−/−^ mice reconstituted with OTII T cells or polyclonal CD4^+^ T cells in the absence of memory CTLs showed a response similar to that of unreconstituted MHCII^−/−^ mice (not shown), suggesting the observed CTL responses were mainly due to memory CTL expansion. Overall, these results indicate the importance of CD4^+^ T helper signals provided during the priming and recall phases for optimal AdV-stimulated memory CTL responses.

## Discussion

In the absence of strong inflammatory signals, CD4^+^ T cells enhance CTL immunity either indirectly by licensing DCs [9]–[11], or directly by interacting with cognate CD8^+^ T cells [14], [38], [39]. Immunity to cancers, allogenic transplantations and autoimmune disorders thus requires CD4^+^ T cells for optimal priming, maintenance and memory responses [1], [8], [38], [40], [41]. Even in some infectious diseases, such as HSV, VSV, viral encephalitis and Vaccinia virus infections, CD4^+^ T cell help is still crucial for the induction of robust primary and functional memory responses [4], [11]–[13], [42] though the viral byproducts (such as DNA or double-stranded RNA) are capable of inducing inflammation. Interestingly, the present study also demonstrates the predominant role for CD4^+^ T cells in multiple phases of AdVova-stimulated OVA-specific CTL responses, including the primary, memory maintenance and recall responses. We also reveal that the differentiation of primary CTLs is considerably impaired in the absence of CD4^+^ T cell-derived CD154 signaling. The CD4^+^ T cell-presented CD154 also appears to be important in various other models, including HSV and Vaccinia virus infections, and cell-based immunogens, where DCs receive poor maturation signals [4], [10], [11], [14], [38]. In the present study, the relatively increased level of primary CTL expansion seen in mice deficient in providing CD4^+^ T cell’s CD154 signal, compared to mice without any CD4^+^ T cell-derived signals, may suggest the involvement of other helper factors in this process. However, a complete failure in antitumor protection during the memory phase in mice missing CD154 signaling indicates the requirement of CD4^+^ T cell’s CD154 for the functional AdV-specific memory CTL development. A similar phenomena with partially functional primary, but completely defective memory CTL responses were also observed in infections caused by LCMV, Pichinde virus and VSV in the absence of CD154 signaling [15], [43]. The presence of pathogen-associated signals, which can induce DC maturation, might explain the modest induction of the CD154-independent primary CTL responses in infections caused by all these viruses, including AdV.

IL-2 signaling not only supports primary CTL expansion, but also helps in the generation of memory CTLs, by providing CTLs with survival advantages. Our observation of the severe impairment of the primary CTL response in the absence of CD4^+^ T cell-derived IL-2 disagrees with a recent report, demonstrating a role of the autocrine action of CD8 CTL-secreted, but not CD4^+^ T cell-secreted IL-2 in the primary and memory responses in Vaccinia virus infection model [44]. These discrepancies are likely to result from differences in the nature of priming stimulus, in doses of the CD4^+^ T cells and in adoptive transfer procedures. However, our data are consistent with previous reports, showing the importance of CD4^+^ T cell-derived IL-2 in priming as well as memory responses [45], [46].

The means by which CD4^+^ T cells provide CD154 and IL-2 signals to optimize primary and memory CTL responses is well described in various models [4], [42], [47]. It was shown that Th1 provide CD154 signals to DCs to induce IL-12 secretion and IL-12, in turn, acts on CD8^+^ T cells to enhance IL-2R expression, thus enabling an efficient utilization of the IL-2 cytokine [4]. It was also shown that DC-stimulated Th1 cells can directly provide CD154, IL-21 and IL-2 signals to CD8^+^ T cells in an Ag-specific manner either in three-cell interactions or in a sequential fashion [14], [38], [39], [47], [48]. A recent study has also shown that CD4^+^ T cells induce DC maturation via CD40:CD154 signaling that enables autocrine IL-2 production in CD8 T cells [4], [42], [47]. Interestingly, cognate CD4^+^ T cells have been shown to modulate CTL responses in a CD154- and IL-2- dependent manner in all these direct and indirect mechanisms. In MHCII^−/−^ mice, the incomplete maturation of DCs without CD4^+^ T cell signals, and/or the absence of direct CD4^+^ T cell signals might have led to poor primary and memory CTL responses. In agreement, we were able to overcome these defectiveness by providing polyclonal or cognate CD4^+^ T cells, which suggests a critical role for CD4^+^ T cells in AdV-triggered CTL induction.

The enhanced recall responses observed upon providing naïve monoclonal or polyclonal, or memory CD4^+^ T cells suggest yet another important role for CD4^+^ T cells in CTL responses. It is currently not clear why memory CTLs also require CD4^+^ T cell environment. It was shown in various models that multiple CD4^+^ T helper signals co-operate in the regulation of recall responses [1], [37], [40], [47]. For instance, treatment with agonistic anti-CD40 mAb and IL-2 is known to decrease the expression of PD-1, a potent T cell inhibitory molecule, on memory CTLs [37]. Consistent with our present observations, memory CD4^+^ T cells have also been shown to enhance the primary responses of naïve and memory CD8^+^ T cells in various models [36]. Moreover, direct interactions between memory CD4^+^ and CD8^+^ T cells, involving CD154 and IL-2 signaling, and leading to enhanced recall responses have also been reported [40].

A considerable attention has been paid to understanding AdV-specific CTL persistency. Due to persistent AdV-carried transgene expression [24], [25], [49], high levels of CTLs maintained for a long period of time might originate from the reactivation of CTLs by APCs or AdV-transduced non-APCs [49]. In this scenario, all these CTLs could exhaust after some time by losing their abilities to secrete cytokines and to protect against viral or tumor challenges, as seen with chronic viral (LCMV and cytomegalovirus) infections [27]. We demonstrate that in the AdV immunization model, a drastic decrease of CTL persistency occurs in the CD4^+^ T cell-deficient environment, which may result from a lack of CD4^+^ T cell-provided signals. It appears that both endured AdV-stimulated and naïve polyclonal CD4^+^ T cells contribute to CTL persistency in WT mice. Indeed, involvement of nonspecific signals from naïve CD4^+^ T cells in increasing fitness and quantities of memory CTLs in acute infections have been reported [50]. Similarly, effects of signals from active polyclonal CD4^+^ T cells on enhancing CTL survival have also been observed [51]. On the other hand, cognate active CD4^+^ T cells were able to support robust CTL priming. Perhaps due to the absence of some specific types of CD4^+^ T helper signals, MHCII^−/−^ animals demonstrate 2 to 3 fold increase in suppressed PD-1^+^ CTLs, when compared to WT mice. Recently, Novy *et al* reported that CD4^+^ T cells enhanced memory CTL survival by providing IL-21 signals in Vaccinia virus model [1], [47]. This possibility could also exist in AdV immunization model, owing to the persistency of AdV-specific CD4^+^ T cells. However, the precise contributions of transgene and non-transgene AdV-stimulated CD4^+^ T cells versus naïve polyclonal CD4^+^ T cells, and associated molecular mechanisms involved in memory CTL survival need further investigation. It has been demonstrated that CD4^+^ T cells can prevent the exhaustion of CD8^+^ T cells during chronic viral and *Plasmodium* parasitic infections [52], [53]. Similarly, exhausted CD8^+^ T cells derived from progressive HIV patients underwent proliferation when co-cultured with CD4^+^ T cells taken from acute HIV patients [52], [54], suggesting that CD4^+^ T helper factors could restore CTL functions in chronic HIV infections. Interestingly, the application of autologous CD4^+^ T cells have also shown to induce prolonged clinical remission in metastatic melanoma patients [55], [56]. Taken together, these results and our observations suggest that the use of supplemental CD4^+^ T cell therapy or of CD4^+^ T helper factors may be beneficial for successful treatment of AdV-immunized patients with chronic infections or malignancies.

Due to their efficiency in inducing strong innate and adaptive immune responses [57], AdVs have recently gained a lot of attention as promising vaccination tools in treating intractable diseases, including cancers [58], [59] and chronic infections [60], [61]. However, AdV-based vaccines often fail to provide a protection in clinical trials, partly due to the chronic nature of pathogenic Ags and the associated imbalance in host immune responses, involving the selective depletion or defectiveness of CD4^+^ T cells [62]–[65]. Our results provide a partial explanation for the mechanism of the failures in AdV-based vaccinations against these intractable diseases, warranting the development of novel modified AdV vaccines.

## Materials and Methods

### Ethics Statement

All the animal experiments were performed as per the guidelines approved by the University Committee on Animal Care and Supply, University of Saskatchewan. Protocol Number: 20100027.

### Reagents, Tumor Cells and Animals

The biotin- or fluorescent-labeled (FITC or PE) Ab specific for CD4, CD44 (IM7), CD62L (MEL-14), CD69 (H1.2F3) and IFN-γ (XMG1.2), and streptavidin-PE Texas Red and streptavidin-PECy5 from BD-Biosciences, and PD-1 (J43) from ebiosciences were purchased. The FITC-anti-CD8 (KT15) and H-2K^b^/OVA_257–264_ tetramer from Beckman Coulter were purchased. The OVAI (OVA_257–264_, SIINFEKL) and OVAII (OVA_265–280_, TEWTSSNVMEERKIKV) peptides were synthesized by Multiple Peptide Systems. The OVA-transfected BL6-10 (BL6-10_OVA_) [39] cell lines were cultured as described previously [14]. The WT C57BL/6J, OVA_323–339_-specific TCR-transgenic OTII, B6.SJL (CD45.1^+^), CD80^−/−^, IL-2^−/−^, CD154^−/−^ and MHCII^−/−^ mice on C57BL/6 background were purchased from Jackson Laboratory or bred in University’s animal resource center. The OTII/B6.1, OTII/CD80^−/−^ and OTII/CD154^−/−^ mice were generated by backcrossing designated KO mice with OTII mice, and tested as described previously [14]. All the animal experiments were performed as per the guidelines approved by the University Committee on Animal Care and Supply.

### Generation of AdVova and Mature DCova

The recombinant AdV-expressing OVA (AdVova) construction and its amplification were previously described [66]. Bone-marrow-derived, OVA-pulsed dendritic cells (DC_OVA_) from C57BL/6 mice were generated by culturing bone marrow cells for 6 days in medium containing IL-4 (20 ng/ml) and GM-CSF (20 ng/ml) and pulsing with 0.1 mg/mL OVA overnight at 37°C as described previously [39].

### Animal Studies

In most experiments, CD4^+^ or CD8^+^ T cells were isolated from splenocytes and/or blood by enriching T lymphocytes in nylon-wool columns (C&A Scientific), and negative selection using anti-CD8 (L3T8) or anti-CD4 (L3T4) paramagnetic beads (DYNAL) as previously described [14]. For immunization or boosting, 1×10^7^ pfu of AdVova was used for i.v. injection. The CD11c^+^ DCs and, in some cases, CD4^+^ T cells devoid of CD11c^+^ CD4^+^ DCs were purified from splenocytes of WT mice as per manufacturer’s instructions (Miltenyl Biotec). In adoptive studies, to understand CD4^+^ T-helper roles, ∼15–20×10^6^ polyclonal CD4^+^ T cells or ∼1.5×10^6^ OTII T cells with or without designated gene deficiency and 0.5–1×10^6^ CD11c^+^ DCs were transferred to MHCII^−/−^ mice one day before immunization. For generating memory CD4^+^ T cells, B6.1/OTII CD4^+^ T cells were stimulated with DCova and resulting Th1 cells were adoptively transferred to congenic WT B6.2 mice. After 45 days, polyclonal CD4^+^ T cells containing CD4^+^ T memory cells were purified and transferred (15–20×10^6^/mouse) to MHCII^−/−^ mice for recall studies. In memory maintenance or recall studies, polyclonal CD8^+^ T cells containing effector or memory CTLs (∼15×10^6^/mouse) were transferred to MHCII^−/−^ mice.

### Primary and Memory CTL Kinetics Study by Tetramer or Intracellular IFN-γ Staining Assays

Following AdVova immunization, the blood and/or spleen samples were collected at different intervals, stained with H-2K^b^/OVA_257–264_ tetramer and FITC-anti-CD8 Ab, and analyzed for CTL proliferation or survival [39]. For intracellular staining, the samples were collected, re-stimulated with OVAI and OVAII and subjected to intracellular IFN-γ assay (BD-Biosciences) for analysis of OVA-specific CD8^+^ and CD4^+^ T cell responses, respectively as described previously [67].

### *In vivo* Cytotoxicity Assay

The targets were prepared as described previously [14], [39] by labeling splenocytes differentially with high (3.0 µM) or low (0.6 µM) concentrations of CFSE and by pulsing with OVAI or Mut1 peptide, respectively, and co-injected i.v. (2×10^6^ cells/mouse) at 1∶1 ratio into immunized or unimmunized mice. Sixteen hours later, the relative proportions of CFSE^high^ and CFSE^low^ cells remaining in the spleens were analyzed by flow cytometry [14], [39].

### Phenotypic Characterization of Memory CTLs and Memory CD4^+^ T Cells

To characterize memory CTLs phenotypically, the blood and spleen samples were collected 75 days after AdVova immunization, and stained with tetramer and anti-PD-1 Ab specific for exhausted CTL phenotype. The percentage of tetramer^+^ CTLs that express PD-1 marker were determined by flow cytometry. To characterize memory CD4^+^ T cells, the blood samples were collected 45 days of adoptive transfer, stained with PE-anti-CD4, FITC-CD45.1 and biotin-conjugated Abs specific for active or memory phenotype. The relative expression of surface markers in comparison to isotype control levels was determined in CD4^+^CD45.1^+^ population.

### Tumor Protection Studies

All the immunized mice were challenged with BL6-10_OVA_ (0.5×10^6^ cells/mice) on 120^th^ day of immunization as shown in Table 1 and monitored for protection up to 24 days or earlier if the mice become moribund as described previously [39]. The tumor grading was done depending on mean numbers of metastatic tumor colonies in lungs: −, no tumors; +, 1–25; ++, 26–50; +++, 51–75; ++++, 76-100; +++++, 101-250; ++++++, >250.

### Statistical Analysis

The statistical analysis were performed using Student’s *t* or Mann-Whitney U test (Graphpad Prism-3.0); *P<0.05 and **P<0.01.
